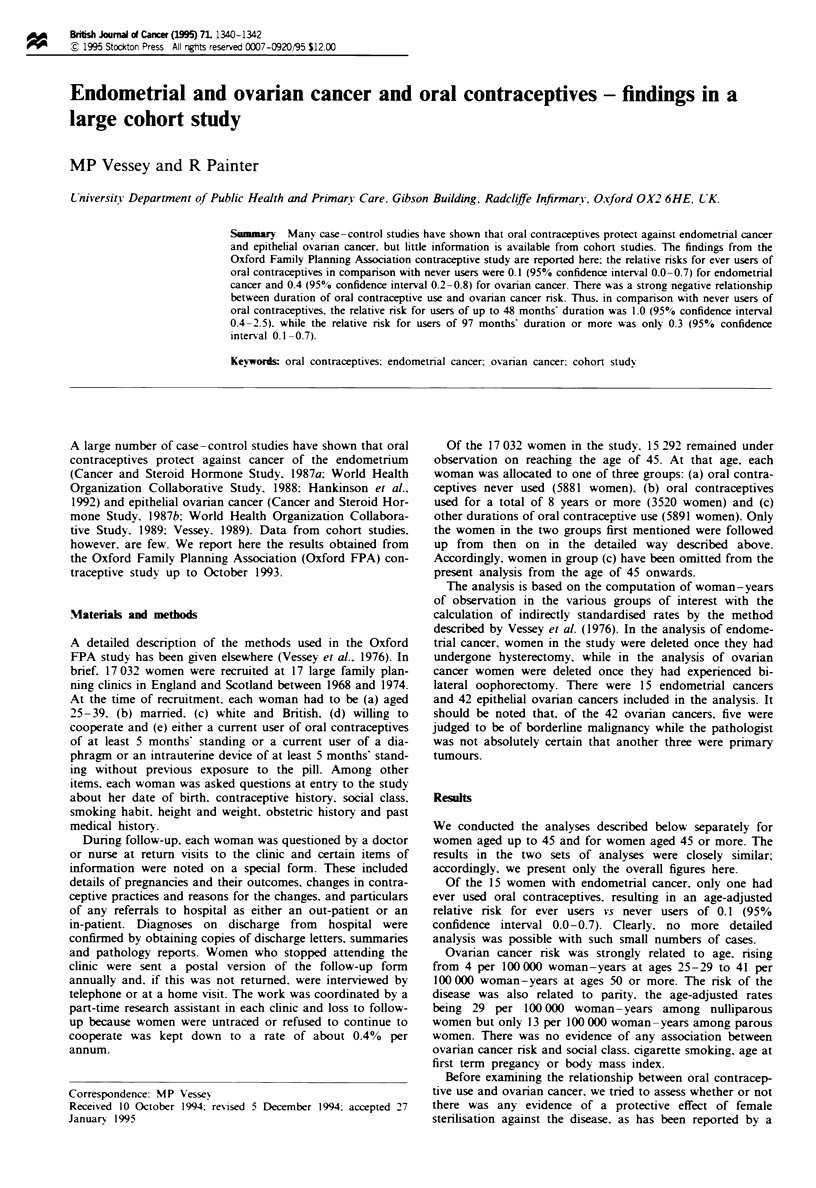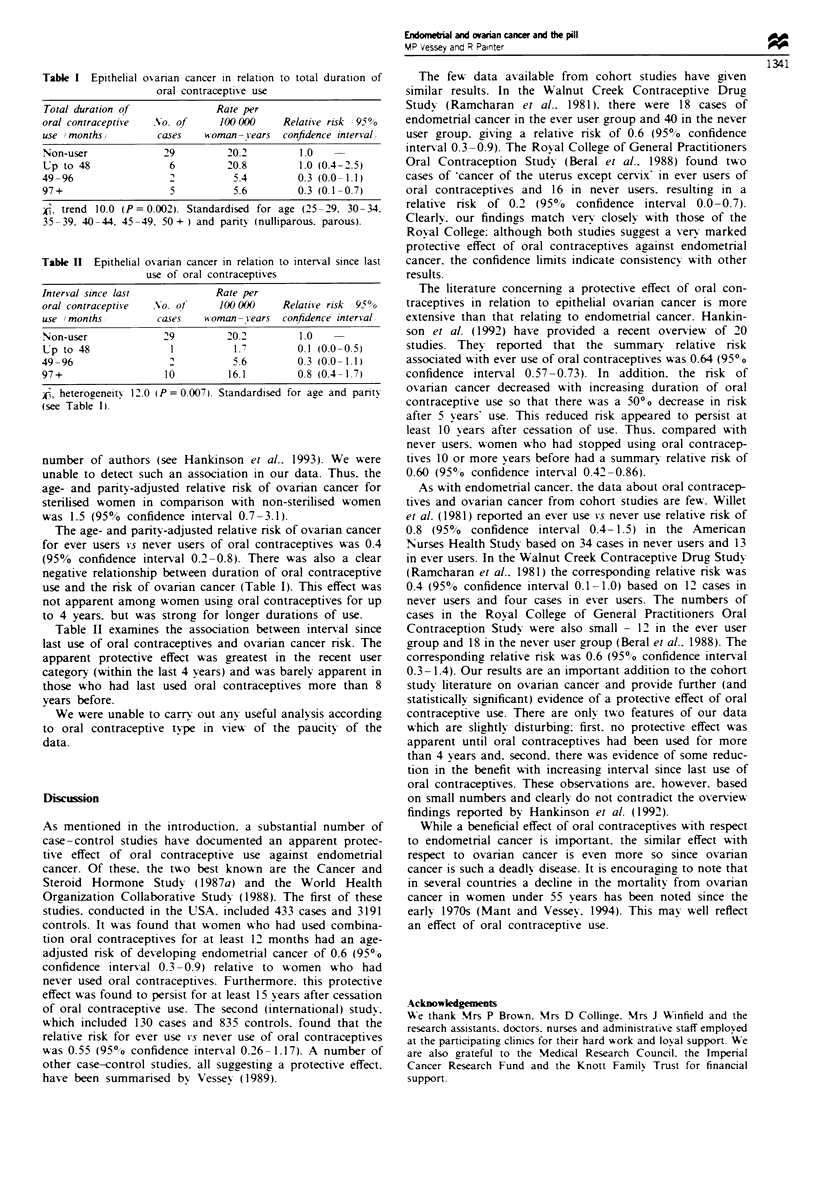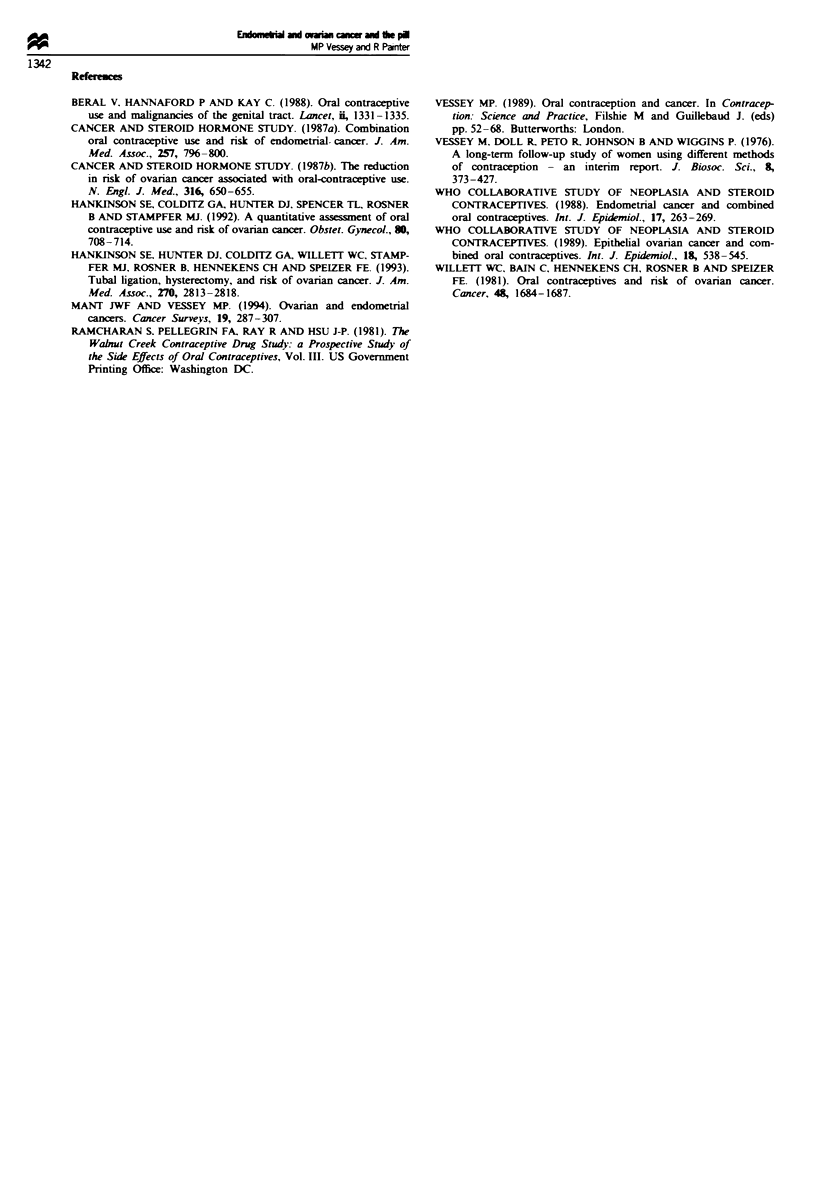# Endometrial and ovarian cancer and oral contraceptives--findings in a large cohort study.

**DOI:** 10.1038/bjc.1995.260

**Published:** 1995-06

**Authors:** M. P. Vessey, R. Painter

**Affiliations:** University Department of Public Health and Primary Care, Radcliffe Infirmary, Oxford, UK.

## Abstract

Many case-control studies have shown that oral contraceptives protect against endometrial cancer and epithelial ovarian cancer, but little information is available from cohort studies. The findings from the Oxford Family Planning Association contraceptive study are reported here; the relative risks for ever users of oral contraceptives in comparison with never users were 0.1 (95% confidence interval 0.0-0.7) for endometrial cancer and 0.4 (95% confidence interval 0.2-0.8) for ovarian cancer. There was a strong negative relationship between duration of oral contraceptive use and ovarian cancer risk. Thus, in comparison with never users of oral contraceptives, the relative risk for users of up to 48 months' duration was 1.0 (95% confidence interval 0.4-2.5), while the relative risk for users of 97 months' duration or more was only 0.3 (95% confidence interval 0.1-0.7).


British Journal of Cancer (1995) 71, 1340-1342

© 1995 Stockton Press All rights reserved 0007-0920/95 $12.00

### Endometrial and ovarian cancer and oral contraceptives – findings in a large cohort study

MP Vessey and R Painter

*University Department of Public Health and Primary Care, Gibson Building, Radcliffe Infirmary, Oxford OX2 6HE, UK.*

**Summary** Many case-control studies have shown that oral contraceptives protect against endometrial cancer and epithelial ovarian cancer, but little information is available from cohort studies. The findings from the Oxford Family Planning Association contraceptive study are reported here; the relative risks for ever users of oral contraceptives in comparison with never users were 0.1 (95% confidence interval 0.0-0.7) for endometrial cancer and 0.4 (95% confidence interval 0.2-0.8) for ovarian cancer. There was a strong negative relationship between duration of oral contraceptive use and ovarian cancer risk. Thus, in comparison with never users of oral contraceptives, the relative risk for users of up to 48 months' duration was 1.0 (95% confidence interval 0.4-2.5), while the relative risk for users of 97 months' duration or more was only 0.3 (95% confidence interval 0.1-0.7).

**Keywords:** oral contraceptives; endometrial cancer; ovarian cancer; cohort study

A large number of case-control studies have shown that oral contraceptives protect against cancer of the endometrium (Cancer and Steroid Hormone Study, 1987*a*; World Health Organization Collaborative Study, 1988; Hankinson *et al.*, 1992) and epithelial ovarian cancer (Cancer and Steroid Hormone Study, 1987*b*; World Health Organization Collaborative Study, 1989; Vessey, 1989). Data from cohort studies, however, are few. We report here the results obtained from the Oxford Family Planning Association (Oxford FPA) contraceptive study up to October 1993.

### Materials and methods

A detailed description of the methods used in the Oxford FPA study has been given elsewhere (Vessey *et al.*, 1976). In brief, 17 032 women were recruited at 17 large family planning clinics in England and Scotland between 1968 and 1974. At the time of recruitment, each woman had to be (a) aged 25-39, (b) married, (c) white and British, (d) willing to cooperate and (e) either a current user of oral contraceptives of at least 5 months' standing or a current user of a diaphragm or an intrauterine device of at least 5 months' standing without previous exposure to the pill. Among other items, each woman was asked questions at entry to the study about her date of birth, contraceptive history, social class, smoking habit, height and weight, obstetric history and past medical history.

During follow-up, each woman was questioned by a doctor or nurse at return visits to the clinic and certain items of information were noted on a special form. These included details of pregnancies and their outcomes, changes in contraceptive practices and reasons for the changes, and particulars of any referrals to hospital as either an out-patient or an in-patient. Diagnoses on discharge from hospital were confirmed by obtaining copies of discharge letters, summaries and pathology reports. Women who stopped attending the clinic were sent a postal version of the follow-up form annually and, if this was not returned, were interviewed by telephone or at a home visit. The work was coordinated by a part-time research assistant in each clinic and loss to follow-up because women were untraced or refused to continue to cooperate was kept down to a rate of about 0.4% per annum.

Correspondence: MP Vessey
Received 10 October 1994; revised 5 December 1994; accepted 27 January 1995

Of the 17 032 women in the study, 15 292 remained under observation on reaching the age of 45. At that age, each woman was allocated to one of three groups: (a) oral contraceptives never used (5881 women), (b) oral contraceptives used for a total of 8 years or more (3520 women) and (c) other durations of oral contraceptive use (5891 women). Only the women in the two groups first mentioned were followed up from then on in the detailed way described above. Accordingly, women in group (c) have been omitted from the present analysis from the age of 45 onwards.

The analysis is based on the computation of woman-years of observation in the various groups of interest with the calculation of indirectly standardised rates by the method described by Vessey *et al.* (1976). In the analysis of endometrial cancer, women in the study were deleted once they had undergone hysterectomy, while in the analysis of ovarian cancer women were deleted once they had experienced bilateral oophorectomy. There were 15 endometrial cancers and 42 epithelial ovarian cancers included in the analysis. It should be noted that, of the 42 ovarian cancers, five were judged to be of borderline malignancy while the pathologist was not absolutely certain that another three were primary tumours.

### Results

We conducted the analyses described below separately for women aged up to 45 and for women aged 45 or more. The results in the two sets of analyses were closely similar; accordingly, we present only the overall figures here.

Of the 15 women with endometrial cancer, only one had ever used oral contraceptives, resulting in an age-adjusted relative risk for ever users *vs* never users of 0.1 (95% confidence interval 0.0-0.7). Clearly, no more detailed analysis was possible with such small numbers of cases.

Ovarian cancer risk was strongly related to age, rising from 4 per 100 000 woman-years at ages 25-29 to 41 per 100 000 woman-years at ages 50 or more. The risk of the disease was also related to parity, the age-adjusted rates being 29 per 100 000 woman-years among nulliparous women but only 13 per 100 000 woman-years among parous women. There was no evidence of any association between ovarian cancer risk and social class, cigarette smoking, age at first term pregancy or body mass index.

Before examining the relationship between oral contraceptive use and ovarian cancer, we tried to assess whether or not there was any evidence of a protective effect of female sterilisation against the disease, as has been reported by a

**Table I** Epithelial ovarian cancer in relation to total duration of oral contraceptive use

| *Total duration of oral contraceptive use (months)* | *No. of cases* | *Rate per 100 000 woman-years* | *Relative risk (95% confidence interval)* |
|---|---|---|---|
| Non-user | 29 | 20.2 | 1.0 — |
| Up to 48 | 6 | 20.8 | 1.0 (0.4-2.5) |
| 49-96 | 2 | 5.4 | 0.3 (0.0-1.1) |
| 97+ | 5 | 5.6 | 0.3 (0.1-0.7) |

$\chi^2_1$, trend 10.0 ($P = 0.002$). Standardised for age (25-29, 30-34, 35-39, 40-44, 45-49, 50+) and parity (nulliparous, parous).

**Table II** Epithelial ovarian cancer in relation to interval since last use of oral contraceptives

| *Interval since last oral contraceptive use (months)* | *No. of cases* | *Rate per 100 000 woman-years* | *Relative risk (95% confidence interval)* |
|---|---|---|---|
| Non-user | 29 | 20.2 | 1.0 — |
| Up to 48 | 1 | 1.7 | 0.1 (0.0-0.5) |
| 49-96 | 2 | 5.6 | 0.3 (0.0-1.1) |
| 97+ | 10 | 16.1 | 0.8 (0.4-1.7) |

$\chi^2_3$, heterogeneity 12.0 ($P = 0.007$). Standardised for age and parity (see Table I).

number of authors (see Hankinson *et al.*, 1993). We were unable to detect such an association in our data. Thus, the age- and parity-adjusted relative risk of ovarian cancer for sterilised women in comparison with non-sterilised women was 1.5 (95% confidence interval 0.7-3.1).

The age- and parity-adjusted relative risk of ovarian cancer for ever users *vs* never users of oral contraceptives was 0.4 (95% confidence interval 0.2-0.8). There was also a clear negative relationship between duration of oral contraceptive use and the risk of ovarian cancer (Table I). This effect was not apparent among women using oral contraceptives for up to 4 years, but was strong for longer durations of use.

Table II examines the association between interval since last use of oral contraceptives and ovarian cancer risk. The apparent protective effect was greatest in the recent user category (within the last 4 years) and was barely apparent in those who had last used oral contraceptives more than 8 years before.

We were unable to carry out any useful analysis according to oral contraceptive type in view of the paucity of the data.

### Discussion

As mentioned in the introduction, a substantial number of case-control studies have documented an apparent protective effect of oral contraceptive use against endometrial cancer. Of these, the two best known are the Cancer and Steroid Hormone Study (1987*a*) and the World Health Organization Collaborative Study (1988). The first of these studies, conducted in the USA, included 433 cases and 3191 controls. It was found that women who had used combination oral contraceptives for at least 12 months had an age-adjusted risk of developing endometrial cancer of 0.6 (95% confidence interval 0.3-0.9) relative to women who had never used oral contraceptives. Furthermore, this protective effect was found to persist for at least 15 years after cessation of oral contraceptive use. The second (international) study, which included 130 cases and 835 controls, found that the relative risk for ever use *vs* never use of oral contraceptives was 0.55 (95% confidence interval 0.26-1.17). A number of other case-control studies, all suggesting a protective effect, have been summarised by Vessey (1989).

The few data available from cohort studies have given similar results. In the Walnut Creek Contraceptive Drug Study (Ramcharan *et al.*, 1981), there were 18 cases of endometrial cancer in the ever user group and 40 in the never user group, giving a relative risk of 0.6 (95% confidence interval 0.3-0.9). The Royal College of General Practitioners Oral Contraception Study (Beral *et al.*, 1988) found two cases of 'cancer of the uterus except cervix' in ever users of oral contraceptives and 16 in never users, resulting in a relative risk of 0.2 (95% confidence interval 0.0-0.7). Clearly, our findings match very closely with those of the Royal College; although both studies suggest a very marked protective effect of oral contraceptives against endometrial cancer, the confidence limits indicate consistency with other results.

The literature concerning a protective effect of oral contraceptives in relation to epithelial ovarian cancer is more extensive than that relating to endometrial cancer. Hankinson *et al.* (1992) have provided a recent overview of 20 studies. They reported that the summary relative risk associated with ever use of oral contraceptives was 0.64 (95% confidence interval 0.57-0.73). In addition, the risk of ovarian cancer decreased with increasing duration of oral contraceptive use so that there was a 50% decrease in risk after 5 years' use. This reduced risk appeared to persist at least 10 years after cessation of use. Thus, compared with never users, women who had stopped using oral contraceptives 10 or more years before had a summary relative risk of 0.60 (95% confidence interval 0.42-0.86).

As with endometrial cancer, the data about oral contraceptives and ovarian cancer from cohort studies are few. Willet *et al.* (1981) reported an ever use *vs* never use relative risk of 0.8 (95% confidence interval 0.4-1.5) in the American Nurses Health Study based on 34 cases in never users and 13 in ever users. In the Walnut Creek Contraceptive Drug Study (Ramcharan *et al.*, 1981) the corresponding relative risk was 0.4 (95% confidence interval 0.1-1.0) based on 12 cases in never users and four cases in ever users. The numbers of cases in the Royal College of General Practitioners Oral Contraception Study were also small - 12 in the ever user group and 18 in the never user group (Beral *et al.*, 1988). The corresponding relative risk was 0.6 (95% confidence interval 0.3-1.4). Our results are an important addition to the cohort study literature on ovarian cancer and provide further (and statistically significant) evidence of a protective effect of oral contraceptive use. There are only two features of our data which are slightly disturbing: first, no protective effect was apparent until oral contraceptives had been used for more than 4 years and, second, there was evidence of some reduction in the benefit with increasing interval since last use of oral contraceptives. These observations are, however, based on small numbers and clearly do not contradict the overview findings reported by Hankinson *et al.* (1992).

While a beneficial effect of oral contraceptives with respect to endometrial cancer is important, the similar effect with respect to ovarian cancer is even more so since ovarian cancer is such a deadly disease. It is encouraging to note that in several countries a decline in the mortality from ovarian cancer in women under 55 years has been noted since the early 1970s (Mant and Vessey, 1994). This may well reflect an effect of oral contraceptive use.

**Acknowledgements**
We thank Mrs P Brown, Mrs D Collinge, Mrs J Winfield and the research assistants, doctors, nurses and administrative staff employed at the participating clinics for their hard work and loyal support. We are also grateful to the Medical Research Council, the Imperial Cancer Research Fund and the Knott Family Trust for financial support.

### References

BERAL V, HANNAFORD P AND KAY C. (1988). Oral contraceptive use and malignancies of the genital tract. *Lancet*, **ii**, 1331-1335.

CANCER AND STEROID HORMONE STUDY. (1987*a*). Combination oral contraceptive use and risk of endometrial cancer. *J. Am. Med. Assoc.*, **257**, 796-800.

CANCER AND STEROID HORMONE STUDY. (1987*b*). The reduction in risk of ovarian cancer associated with oral-contraceptive use. *N. Engl. J. Med.*, **316**, 650-655.

HANKINSON SE, COLDITZ GA, HUNTER DJ, SPENCER TL, ROSNER B AND STAMPFER MJ. (1992). A quantitative assessment of oral contraceptive use and risk of ovarian cancer. *Obstet. Gynecol.*, **80**, 708-714.

HANKINSON SE, HUNTER DJ, COLDITZ GA, WILLETT WC, STAMPFER MJ, ROSNER B, HENNEKENS CH AND SPEIZER FE. (1993). Tubal ligation, hysterectomy, and risk of ovarian cancer. *J. Am. Med. Assoc.*, **270**, 2813-2818.

MANT JWF AND VESSEY MP. (1994). Ovarian and endometrial cancers. *Cancer Surveys*, **19**, 287-307.

RAMCHARAN S, PELLEGRIN FA, RAY R AND HSU J-P. (1981). *The Walnut Creek Contraceptive Drug Study: a Prospective Study of the Side Effects of Oral Contraceptives*, Vol. III. US Government Printing Office: Washington DC.

VESSEY MP. (1989). Oral contraception and cancer. In *Contraception: Science and Practice*, Filshie M and Guillebaud J. (eds) pp. 52-68. Butterworths: London.

VESSEY M, DOLL R, PETO R, JOHNSON B AND WIGGINS P. (1976). A long-term follow-up study of women using different methods of contraception - an interim report. *J. Biosoc. Sci.*, **8**, 373-427.

WHO COLLABORATIVE STUDY OF NEOPLASIA AND STEROID CONTRACEPTIVES. (1988). Endometrial cancer and combined oral contraceptives. *Int. J. Epidemiol.*, **17**, 263-269.

WHO COLLABORATIVE STUDY OF NEOPLASIA AND STEROID CONTRACEPTIVES. (1989). Epithelial ovarian cancer and combined oral contraceptives. *Int. J. Epidemiol.*, **18**, 538-545.

WILLETT WC, BAIN C, HENNEKENS CH, ROSNER B AND SPEIZER FE. (1981). Oral contraceptives and risk of ovarian cancer. *Cancer*, **48**, 1684-1687.